# Mortality among US employees of a large computer manufacturing company: 1969–2001

**DOI:** 10.1186/1476-069X-5-30

**Published:** 2006-10-19

**Authors:** Richard W Clapp

**Affiliations:** 1 Department of Environmental Health, Boston University School of Public Health, 715 Albany Street, Boston, MA, USA 02118

## Abstract

**Background:**

Previous studies suggested increased cancer incidence and mortality in workers exposed to solvents and other chemicals in computer manufacturing jobs. Most previous studies were of small cohorts and findings were inconsistent. A lawsuit involving a large U.S. company produced a data file for analysis. This study sought to elucidate patterns of mortality in workers who were engaged manufacturing computers and related electronic components in the largest database available to date.

**Methods:**

A proportional mortality and proportional cancer mortality analysis of deaths in eligible workers between 1969 and 2001 was carried out, with U.S. population mortality data as the standard for comparison. Mortality and work history data was from corporate mortality and work history files produced during litigation and standard U.S. and state mortality files. The study base comprised 31,941 decedents who died between 1969 and 2001, who had worked for at least five years and whose death information was collected in the corporate mortality file. Proportional mortality ratios (PMRs) and Proportional Cancer Mortality Ratios (PCMRs) and their 95% confidence intervals were computed for 66 causes of death in males and females.

**Results:**

PMRs for all cancers combined were elevated in males (PMR = 107; 95% CI = 105–109) and females (PMR = 115; 95% CI = 110–119); several specific cancers and other causes of death were also significantly elevated in both males and females. There were reduced deaths due to non-malignant respiratory disease in males and females and heart disease in females; several specific cancers and other causes of death were significantly reduced in both males and females. Proportional cancer mortality ratios (PCMRs) for brain and central nervous system cancer were elevated (PCMR = 166; 95% CI = 129–213), kidney cancer (PCMR = 162; 95% CI = 124–212), melanoma of skin (PCMR = 179; 95% CI = 131–244) and pancreatic cancer (PCMR = 126; 95% CI = 101–157) were significantly elevated in male manufacturing workers. Kidney cancer (PCMR = 212; 95% CI = 116–387) and cancer of all lymphatic and hematopoietic tissue (PCMR = 162; 95% CI = 121–218) were significantly elevated in female manufacturing workers.

**Conclusion:**

Mortality was elevated due to specific cancers and among workers more likely to be exposed to solvents and other chemical exposures in manufacturing operations. Due to lack of individual exposure information, no conclusions are made about associations with any particular agent.

## Background

In 2002, plaintiffs' attorneys in a lawsuit obtained computerized files of deaths and work histories for US employees of a large company which manufactured electronic office equipment; mainframe and personal computers; computer parts, components, and accessories; and software products. The files contained data on employees who had qualified for pension and death benefits and died in the US between the years 1969 and 2001. A unique identifier was included in both files so that death and work history data for individual decedents could be linked.

Few studies of workers in computer manufacturing and semiconductor fabrication are available in the published literature. A study of workers at a Scottish semiconductor plant was conducted by the government occupational health authority in 2001 [1,2]. This study showed statistically significant excesses of lung, stomach, and breast cancers among females and an excess of brain cancer among males; however, the number of employees in this plant was relatively small and the number of cases of cancer was limited. Recently, an update of a UK semiconductor workers study was published which was also small and had few significant findings with regard to cancer. A previous excess incidence of melanoma persisted in the update, along with excess rectal and pancreatic cancer in females [3]. Two studies of workers who manufactured main boards and integrated circuits in an electronics company in Taiwan were published [4,5]. In the cancer incidence analysis there was a significant excess of breast cancer in female electronics company workers when compared to two other groups of industrial workers [5]. An analysis of mortality in three U.S. semiconductor and storage device-manufacturing plants identified significant excess central nervous system cancer in one plant and excess prostate cancer in workers in another plant [6].

There are a variety of exposures to chemicals, metals (especially arsenic, nickel and chromium), and electromagnetic fields (especially ultraviolet light, radiofrequency, and, in one process, x-ray radiation) in computer and semiconductor manufacturing [7]. There have been many studies of workers with these exposures in other industries. For example, trichloroethylene is used in many industries and there is a large literature on its health effects [8]. Similarly, the health effects of methylene chloride, isopropyl alcohol, methyl ethyl ketone, Freon, as well as metals and electromagnetic radiation have been studied in other groups of workers and in experimental animals [9,10]. Furthermore, various US and international agencies have classified several of these chemicals as to their carcinogenicity [11,12].

Based upon the available literature, workers exposed to the chemicals and processes in computer and semiconductor manufacturing would be expected to have elevated mortality from non-Hodgkin's lymphoma [8,11], brain cancer [9], kidney cancer [8,11], lung cancer [2,11], and breast [5] cancer. Additionally, exposures in the computer and semiconductor manufacturing industries are complex and have changed as new processes and materials were introduced. Thus, the mortality experience of computer and semiconductor manufacturing workers would be expected to vary over time due to changing exposures.

We evaluated these expectations and examined the mortality among US employees of a large computer manufacturing company by calculating overall and sex-, age-, and time-specific proportional mortality ratios (PMRs) and proportional cancer mortality ratios (PCMRs). We performed analyses for all eligible decedents and the subgroup of decedents who were ever employed 30 days or more in manufacturing.

## Methods

The context of this analysis is unusual because the data were obtained as part of litigation and the company's attorneys resisted making the files available. A court order forced the production of the files, and minimal documentation was provided about the content and coding of the files. Due to the context in which we received these data, we were not able to obtain additional documentation, access the original or complementary data, or contact company personnel knowledgeable about the data. Since we could not resolve problems in the data, we spent considerable time developing logical rules for handling incomplete and apparently erroneous information. These constraints limited the data available for subgroup analyses.

### Corporate Mortality File

At the time it was provided to plaintiffs' attorneys, the IBM Corporate Mortality File (CMF) maintained by the company had 33,730 records. These records were for decedents who had been employed for five years or more, who were actively employed or receiving retirement or disability benefits at time of death, and whose families had filed a claim for death benefits. CMF data required for analysis were the identifier, sex, birth date, death date, and underlying cause of death.

We reviewed the numbers of deaths by year of death. Based on the apparent degree of completeness of the CMF by year of death, we defined the study period as 1969–2001. We excluded duplicate records and records for which the sex, birth date, and/or death date remained unknown or discrepant. Of the 33,730 records in the CMF, 1,456 records were excluded for these reasons. Processing of the work history data (see below) identified 20 additional CMF records with discrepant birth and/or death dates and these also were excluded. We then excluded 313 CMF records for which the year of death was before 1969 or after 2001.

The final number of CMF records used for analysis was 31,941 and comprised 27,272 males and 4,669 females.

### Work History Data

The work history data set had a total of 586,258 records representing 18,928 decedents. Work history data required for analysis were the identifier, effective date, position code, location code, and text notes indicating a key employment event such as leave of absence, return from leave of absence, separation, retirement, or death.

We added the birth and death dates from the CMF to the work history data set. We then identified irrelevant, missing, invalid, and inconsistent work history data to the extent feasible.

We deleted all work history records for decedents that were excluded from the CMF (see above). Likewise, we deleted all work history records with irrelevant, missing, invalid or inconsistent data. Upon completion of these steps, the work history data set included a total of 375,563 records representing 10,219 decedents. There did not appear to be any systematic pattern in the missing data that would produce bias in the analysis of this subset of decedents.

### Cause of Death Coding

The underlying causes of death were coded to the Ninth Revision of the International Classification of Diseases (ICD 9).

### Comparison Population

We selected the US as the most appropriate comparison population for the overall CMF analyses and we used it as the comparison population for the exposure subgroup analyses for consistency. We obtained cause-, sex-, age-, and time-specific mortality data, adjusted to ICD 9, for the US comparison population from the Mortality and Population Data System (MPDS) at the University of Pittsburgh [13]. We used default categories for sex, age, and time, and default and "custom" categories for cause of death. The mortality data were not categorized by race because race was not known for the decedents included in the CMF.

### Analysis

We analyzed the data with the Occupational Cohort Mortality Analysis Program (i.e., OCMAP-PLUS version 3.10) software package from the University of Pittsburgh [13]. In addition to the 63 OCMAP-PLUS default cause of death categories, we defined the following six "custom" cause of death categories: non-Hodgkin's lymphoma, multiple myeloma, diseases of the nervous system, multiple sclerosis, Parkinson's disease, and amyotrophic lateral sclerosis.

Standardized proportional mortality ratios (PMRs) were calculated from the ratio of the observed number of deaths due to a particular cause among the workers to the expected number of deaths based on the age-, sex-, time-, and cause-specific proportional mortalities of the US population. The expected number of deaths was computed by multiplying the proportional mortalities of the US population by the total number of deaths for the workers. The PMRs were standardized on age, sex, and time. Standardized proportional cancer mortality ratios (PCMRs) also were calculated. For the PCMRs, site-specific cancer proportional mortality was computed as a proportion of all cancer mortality. The underlying cause of death was used for calculation of the PMRs and PCMRs. Workers with an unknown cause of death were included in the "all causes of death" and "unknown causes" categories. The associated 95% Poisson confidence intervals (95% CIs) for the PMRs and PCMRs were calculated as well.

### Exposure Subgroups

We defined the following exposure subgroups for both men and women:

• ever worked in manufacturing for 30 days or more;

• ever worked at a selected California, Minnesota, New York and Vermont location for 30 days or more.

The latter four state sub-groups were defined for analyses of specific plants of interest in the litigation (data not shown).

## Results

The number of decedents and the percent of decedents with available and usable work history information are shown in Table 1 by year of death and sex. Of the 27,272 male decedents, 9,219 (33.8%) had work histories. For the 4,669 female decedents, 1,000 (21.4%) had work histories. In general, work history information was most complete for the years 1980 – 1999 and was more complete for men than women. There were 3,652 male and 723 female decedents whose work history information indicated that they worked 30 days or more in manufacturing.

**Table 1:** Number and Percent of Decedents with Work History by Year of Death

	Males			Females		
Year	Number Of Deaths	Number With Work History	Percent With Work History	Number Of Deaths	Number With Work History	Percent With Work History

1969	241	1	0.4	28	0	0.0
1970–1974	1,758	6	0.3	169	0	0.0
1975–1979	2,246	135	6.0	329	11	3.3
1980–1984	3,135	1,100	35.1	520	108	20.8
1985–1989	4,325	2,089	48.3	754	252	33.4
1990–1994	5,370	2,520	46.9	909	255	28.1
1995–1999	7,386	3,097	41.9	1,377	343	24.9
2000–2001	2,811	271	9.6	583	31	5.3
TOTAL	27,272	9,219	33.8	4,669	1,000	21.4

The all cause average age at death was 64.5 years for male decedents and 63.4 for female decedents. The all cancer average age at death was 63.9 years for male decedents and 59.5 for female decedents.

Cause-specific PMRs and 95% CIs for male decedents in the CMF were calculated and the results for major causes of death are summarized in Table 2. There were 27,272 deaths from all causes. The PMR for all cancers was significantly greater than 100. There were 7,697 cancer deaths and 7,206 were expected (PMR = 107; 95% CI = 105, 109). This excess was due to statistically significant excesses for cancer of the digestive organs and peritoneum, testicular and other male genital cancer, kidney cancer, malignant melanoma of the skin, brain and central nervous system cancer, thyroid and other endocrine cancer, and lymphatic and hematopoietic cancer.

**Table 2:** Cause-Specific Proportional Mortality Ratios (PMRs) for Males

Cause Of Death	Observed	Expected	PMR	95% CI	
All Causes of Death	27,272	27,272.00	100.0	---	---
All Malignant Neoplasms	7,697	7,206.21	106.8†	104.8	108.8
Cancer of Pancreas	441	351.78	125.4†	114.3	137.5
Cancer of Kidney	265	181.06	146.4†	129.9	164.9
Cancer of Central Nervous System	355	185.11	191.8†	173.2	212.3
Malignant Melanoma of Skin	221	115.38	191.5†	168.3	218.0
Non-Hodgkin's Lymphoma	395	262.59	150.4†	136.5	165.8
All Heart Disease	9,044	9,228.90	98.0†	96.4	99.6
Non-malignant Respiratory Disease	1636	2,131.06	76.8†	73.3	80.4
All External Causes of Death	1,809	2,440.60	74.1†	71.3	77.0

Male Corporate Mortality File deaths compared to male U.S. deaths due to all causes, 1969–2001.

† = p < 0.05

In addition, statistically significant excesses were observed for benign neoplasms, nervous system diseases, ischemic heart disease, and all other causes of death, although the excess for ischemic heart disease was quite small. Statistically increased PMRs for multiple sclerosis, Parkinson's disease, and amyotrophic lateral sclerosis contributed to the excess for diseases of the nervous system. There also were deficits of several causes of death as estimated by the PMR method.

In Table 3, we present the cause-specific PCMRs and 95% CIs for males in the CMF. There were 7,697 deaths from all cancers in this group. Except for testicular and other male genital organ cancer, all of the significantly increased PMRs for specific cancers also had significantly increased PCMRs, but the magnitudes of the PCMRs were less than those for the PMRs. The testicular cancer PMR was statistically significantly increased, whereas the PCMR was not (PCMR = 91; 95% CI = 63, 130).

**Table 3:** Cause-Specific Proportional Cancer Mortality Ratios (PCMRs) for Males

Cause Of Death	Observed	Expected	PCMR	95% CI	
All Malignant Neoplasms	7,697	7,697.00	100.0	---	---
Cancer of Buccal Cavity & Pharynx	126	189.63	66.4†	56.0	78.8
Cancer of Digestive Organs & Peritoneum	2,025	1,868.27	108.4†	104.4	112.6
Cancer of Esophagus	239	243.68	98.1	86.6	111.1
Cancer of Stomach	225	239.30	94.0	82.7	106.9
Cancer of Large Intestine	780	628.97	124.0†	116.0	132.6
Cancer of Rectum	135	130.29	103.6	87.7	122.5
Cancer of Biliary Passages & Liver	160	200.03	80.0†	68.7	93.2
Cancer of Pancreas	441	372.21	118.5†	108.2	129.8
Cancer of All Other Digestive Organs	45	47.42	94.9	70.9	127.0
Cancer of Respiratory System	2,220	2,837.10	78.2†	75.7	80.8
Cancer of Larynx	44	98.28	44.8†	33.6	59.6
Cancer of Trachea, Bronchus, & Lung	2,151	2,711.92	79.3†	76.7	82.0
Cancer of All Other Respiratory Organs	25	26.90	92.9	62.8	137.4
Cancer of Breast	12	9.51	126.1	71.8	221.8
Cancer of Prostate	648	603.95	107.3	99.9	115.3
Cancer of Testes & Other Male Genital Organs	28	30.85	90.8	63.2	130.3
Cancer of Kidney	265	194.74	136.1†	120.9	153.2
Cancer of Bladder & Other Urinary Organs	194	172.61	112.4	97.8	129.1
Malignant Melanoma of Skin	221	138.23	159.9†	140.6	181.8
Cancer of Eye	2	3.96	50.5	13.0	196.5
Cancer of Brain and Other Central Nervous System	355	219.24	161.9†	146.4	179.1
Cancer of Thyroid & Other Endocrine Glands	43	22.50	191.1†	142.5	256.2
Cancer of Bone	20	20.44	97.8	63.3	151.2
Cancer of All Lymphatic, Hematopoietic Tissue	938	761.22	123.2†	116.1	130.8
Lymphosarcoma & Reticulosarcoma	65	49.84	130.4†	102.6	165.8
Hodgkin's Disease	41	47.21	86.8	64.4	117.1
Leukemia & Aleukemia	327	288.48	113.4†	102.0	126.0
Cancer of All Other Lymphopoietic Tissue	505	375.72	134.4†	123.5	146.3
Non-Hodgkin's Lymphoma	395	291.46	135.5†	123.1	149.2
Multiple Myeloma	169	126.93	133.1†	114.7	154.5
All Other Malignant Neoplasms	598	631.10	94.8	87.8	102.3

Male Corporate Mortality File deaths compared to male U.S. cancer deaths, 1969–2001.

† = p < 0.05

The cause-specific PMRs and 95% CIs for the 4,669 female decedents in the CMF also were calculated and the results for major causes of death are summarized in Table 4. The PMR for all cancers was significantly greater than 100 for women as well. There were 1,667 cancer deaths and 1,454 were expected (PMR = 115; 95% CI = 110, 119). This excess was due to statistically significant excesses for respiratory system cancer, breast cancer, cancer of other female genital organs, brain and central nervous system cancer, and lymphatic and hematopoietic cancer. A statistically significant excess also was observed for all other causes of death. As in males, there were statistically significant deficits for several causes as estimated by the PMR method.

**Table 4:** Cause-Specific Proportional Mortality Ratios (PMRs) for Females

Cause Of Death	Observed	Expected	PMR	95% CI	
All Causes of Death	4,669	4,669.00	100.0	---	---
All Malignant Neoplasms	1,667	1,454.39	114.6†	110.3	119.1
Cancer of Pancreas	71	66.29	107.1	85.0	134.9
Cancer of Breast	418	306.15	136.5†	124.6	149.6
Cancer of Kidney	31	22.91	135.3	95.4	192.0
Cancer of Central Nervous System	51	38.53	132.4†	100.8	173.8
Malignant Melanoma of Skin	26	19.61	132.6	90.5	194.3
Non-Hodgkin's Lymphoma	70	50.16	139.5†	110.7	176.0
All Heart Disease	998	1,229.92	81.1†	77.1	85.5
Non-malignant Respiratory Disease	279	348.41	80.1†	71.6	89.6
All External Causes of Death	288	341.75	84.3†	76.2	93.2

Female Corporate Mortality File deaths compared to female U.S. deaths due to all causes, 1969–2001.

† = p < 0.05

In Table 5, the cause-specific PCMRs and 95% CIs for females in the CMF are shown. There were 1,667 deaths from all cancers in this group. The PCMR for breast cancer (PCMR = 115; 95% CI = 106, 125) remained significantly elevated, while the PCMRs for several other cancers were no longer elevated compared to their respective PMRs. These cancers included the following: respiratory system cancer; cancer of the trachea, bronchus, and lung; cancer of other female genital organs; brain and central nervous system cancer; lymphatic and hematopoietic cancer; leukemia and aleukemia; non-Hodgkin's lymphoma; and all other lymphatic and hematopoietic cancer. Furthermore, the PCMRs for cancer of the buccal cavity and pharynx, and cancer of the digestive organs and peritoneum were significantly decreased compared to the corresponding PMRs.

**Table 5:** Cause-Specific Proportional Cancer Mortality Ratios (PCMRs) for Females

Cause Of Death	Observed	Expected	PCMR	95% CI	
All Malignant Neoplasms	1,667	1,667.00	100.0	---	---
Cancer of Buccal Cavity & Pharynx	9	19.08	47.2†	25.0	88.9
Cancer of Digestive Organs & Peritoneum	289	323.00	89.5†	80.8	99.1
Cancer of Esophagus	13	16.13	80.6	47.0	138.3
Cancer of Stomach	34	32.53	104.5	74.9	145.7
Cancer of Large Intestine	120	133.00	90.2	76.1	107.0
Cancer of Rectum	18	22.61	79.6	50.4	125.8
Cancer of Biliary Passages & Liver	23	33.92	67.8	45.4	101.3
Cancer of Pancreas	71	72.74	97.6	77.8	122.5
Cancer of All Other Digestive Organs	10	11.11	90.0	48.6	166.9
Cancer of Respiratory System	376	365.12	103.0	94.3	112.4
Cancer of Larynx	1	5.77	17.3†	3.1	97.4
Cancer of Trachea, Bronchus, & Lung	373	355.71	104.9	96.0	114.6
Cancer of All Other Respiratory Organs	2	3.64	55.0	14.1	215.0
Cancer of Breast	418	362.40	115.3†	106.1	125.4
All Uterine Cancers	53	94.10	56.3†	43.6	72.8
Cancer of Cervix Uteri	23	55.35	41.6†	28.3	61.0
Cancer of Other Female Genital Organs	116	104.90	110.6	92.7	131.9
Cancer of Kidney	31	25.81	120.1	84.7	170.2
Cancer of Bladder & Other Urinary Organs	13	15.55	83.6	48.8	143.4
Malignant Melanoma of Skin	26	24.47	106.2	72.7	155.4
Cancer of Eye	0	.86	---	---	---
Cancer of Brain and Other Central Nervous System	51	46.92	108.7	83.0	142.3
Cancer of Thyroid & Other Endocrine Glands	10	6.64	150.6	81.5	278.3
Cancer of Bone	2	4.13	48.5	12.6	186.6
Cancer of All Lymphatic, Hematopoietic Tissue	167	149.40	111.8	96.9	129.0
Lymphosarcoma & Reticulosarcoma	8	8.70	92.0	46.2	183.0
Hodgkin's Disease	8	9.79	81.7	41.5	161.0
Leukemia & Aleukemia	64	56.27	113.7	89.6	144.4
Cancer of All Other Lymphopoietic Tissue	87	74.65	116.5	95.0	143.0
Non-Hodgkin's Lymphoma	70	57.18	122.4	97.3	154.0
Multiple Myeloma	25	25.20	99.2	67.3	146.3
All Other Malignant Neoplasms	106	125.53	84.4	70.3	101.4

Female Corporate Mortality File deaths compated to female U.S. cancer deaths, 1969–2001.

† = p < 0.05

In Table 6, PCMRs are presented for males in the CMF who worked in manufacturing for 30 days or more. There were 1,180 deaths from all cancers in this group. In comparison to the PMRs, only the PCMRs for pancreatic cancer (PCMR = 126; 95% CI = 101, 157), kidney cancer (PCMR = 162; 95% CI = 124, 212), malignant melanoma of the skin (PCMR = 179; 95% CI = 131, 244), and brain and central nervous system cancer (PCMR = 166; 95% CI = 129, 213) remained statistically significant.

**Table 6:** Cause-Specific Proportional Cancer Mortality Ratios (PCMRs) for Male Manufacturing Workers

Cause Of Death	Observed	Expected	PCMR	95% CI	
All Malignant Neoplasms	1,180	1,180.00	100.0	---	---
Cancer of Buccal Cavity & Pharynx	20	30.79	64.9†	42.3	99.7
Cancer of Digestive Organs & Peritoneum	303	287.11	105.5	95.7	116.4
Cancer of Esophagus	38	41.54	91.5	67.0	125.0
Cancer of Stomach	35	35.12	99.6	71.9	138.1
Cancer of Large Intestine	91	93.08	97.8	80.3	119.1
Cancer of Rectum	27	19.38	139.3	96.0	202.1
Cancer of Biliary Passages & Liver	29	32.90	88.1	61.6	126.1
Cancer of Pancreas	73	57.99	125.9†	100.7	157.4
Cancer of All Other Digestive Organs	10	6.99	143.1	77.4	264.7
Cancer of Respiratory System	389	464.42	83.8†	77.6	90.4
Cancer of Larynx	7	16.29	43.0†	21.0	87.8
Cancer of Trachea, Bronchus, & Lung	378	444.16	85.1†	78.6	92.1
Cancer of All Other Respiratory Organs	4	3.96	100.9	37.9	268.3
Cancer of Breast	2	1.42	140.9	35.5	559.2
Cancer of Prostate	77	68.98	111.6	90.1	138.3
Cancer of Testes & Other Male Genital Organs	2	2.99	66.8	17.1	260.9
Cancer of Kidney	51	31.49	162.0†	123.9	211.8
Cancer of Bladder & Other Urinary Organs	23	22.36	102.9	68.7	154.2
Malignant Melanoma of Skin	38	21.23	179.0†	131.4	243.9
Cancer of Eye	0	.58	---	---	---
Cancer of Brain and Other Central Nervous System	57	34.45	165.5†	128.6	212.9
Cancer of Thyroid & Other Endocrine Glands	4	3.34	119.9	45.1	318.6
Cancer of Bone	2	2.56	78.1	19.7	308.9
Cancer of All Lymphatic, Hematopoietic Tissue	115	109.46	105.1	88.4	124.9
Lymphosarcoma & Reticulosarcoma	8	5.14	155.7	78.6	308.4
Hodgkin's Disease	2	4.89	40.9	10.9	153.9
Leukemia & Aleukemia	35	39.94	87.6	63.4	121.2
Cancer of All Other Lymphopoietic Tissue	70	59.49	117.7	93.7	147.8
Non-Hodgkin's Lymphoma	56	44.42	126.1	97.6	162.8
Multiple Myeloma	21	19.55	107.4	70.3	164.1
All Other Malignant Neoplasms	97	98.93	98.1	81.1	118.6

Male Corporate Mortality File manufacturing worker deaths compared to male U.S. cancer deaths, 1969–2001.

† = p < 0.05

In Table 7, PCMRs are presented for female manufacturing workers. All cancers accounted for 302 deaths in this group. The PCMRs for kidney cancer and the other lymphatic and hematopoietic cancers (i.e., leukemia and aleukemia and all other lymphatic and hematopoietic cancer) were significantly increased in this subgroup.

**Table 7:** Cause-Specific Proportional Cancer Mortality Ratios (PCMRs) for Female Manufacturing Workers

Cause Of Death	Observed	Expected	PCMR	95% CI	
All Malignant Neoplasms	302	302.00	100.0	---	---
Cancer of Buccal Cavity & Pharynx	1	3.46	28.9	4.6	179.8
Cancer of Digestive Organs & Peritoneum	49	54.62	89.7	69.7	115.4
Cancer of Esophagus	3	3.01	99.7	32.4	307.3
Cancer of Stomach	9	5.26	171.0	90.2	324.2
Cancer of Large Intestine	17	22.06	77.1	48.9	121.6
Cancer of Rectum	3	3.77	79.6	25.9	244.6
Cancer of Biliary Passages & Liver	2	5.98	33.4	9.1	123.3
Cancer of Pancreas	14	12.69	110.3	66.1	183.9
Cancer of All Other Digestive Organs	1	1.84	54.4	7.9	372.2
Cancer of Respiratory System	65	73.66	88.2	71.6	108.7
Cancer of Larynx	1	1.17	85.3	12.1	601.6
Cancer of Trachea, Bronchus, & Lung	64	71.86	89.1	72.1	110.0
Cancer of All Other Respiratory Organs	0	.63	---	---	---
Cancer of Breast	65	67.00	97.0	78.5	119.9
All Uterine Cancers	8	16.05	49.9†	25.8	96.3
Cancer of Cervix Uteri	3	9.25	32.4†	11.4	92.4
Cancer of Other Female Genital Organs	25	19.00	131.6	90.1	192.0
Cancer of Kidney	10	4.71	212.2†	116.4	386.9
Cancer of Bladder & Other Urinary Organs	1	2.31	43.2	6.5	287.6
Malignant Melanoma of Skin	2	4.21	47.5	12.4	181.5
Cancer of Eye	0	.14	---	---	---
Cancer of Brain and Other Central Nervous System	10	8.40	119.1	64.8	219.0
Cancer of Thyroid & Other Endocrine Glands	0	1.10	---	---	---
Cancer of Bone	0	.60	---	---	---
Cancer of All Lymphatic, Hematopoietic Tissue	40	24.64	162.4†	121.2	217.5
Lymphosarcoma & Reticulosarcoma	0	1.12	---	---	---
Hodgkin's Disease	3	1.26	237.7	80.4	702.6
Leukemia & Aleukemia	15	9.05	165.8†	101.6	270.8
Cancer of All Other Lymphopoietic Tissue	22	13.21	166.5†	111.2	249.4
Non-Hodgkin's Lymphoma	14	9.74	143.8	86.2	240.0
Multiple Myeloma	8	4.50	177.8	90.3	350.2
All Other Malignant Neoplasms	26	22.10	117.6	81.3	170.2

Female Corporate Manufacturing File manufacturing worker deaths compared to female U.S. cancer deaths, 1969–2001.

† = p < 0.05

**Figure 1:**
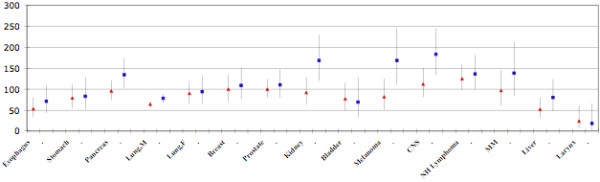
**Comparison of SMRs and PCMRs**. Comparison of SMRs (triangles) for selected cancers from Table 2 in ref. 6 with PCMRs (squares) for manufacturing plant decedents. The vertical lines are the Upper and Lower 95% Confidence Limits of the SMRs and PCMRs. All data are for males and females combined except for prostate, breast and lung cancer; SMRs are for the years 1965–1999 and for those who died 15 or more years since first employment. PCMRs are for the years 1969–2001 and for those who died ten or more years since first employment.

In Fig. 1, we compare the PCMRs for males and females combined who worked 30 days or more in manufacturing in the CMF analyses with the SMRs from the combined CA, NY and VT plants analyzed by other authors [[6], Table 2]. Although there is considerable overlap in the two studies, the time periods, numbers of deaths, and sources of information are different, so only a graphical comparison of results for specific cancers is presented.

## Discussion

We found an overall excess mortality for all cancer in males and females in the entire corporate mortality data. The PMRs for all sites were significantly elevated when comparing the computer company workers to the US population experience during the time period 1969–2001. In addition, there was excess mortality in both males and females due to several types of cancer.

Because of the potential for lowered mortality due to major causes such as ischemic heart disease or respiratory disease, we calculated PCMRs, which focused specifically on cancer mortality. The PCMR is less susceptible to distortion by the "healthy worker effect," and focuses on the health outcome of most interest in these analyses. The results of the PCMR analyses in males were for the most part quite similar to the PMR results for the same cancers. For cancer of the testis, however, the PCMR was 91 (95% CI 63,130) as compared to a PMR of 146. In females, the PCMR for breast cancer was significantly elevated, while the PCMRs for several other cancers were not, compared to their respective PMRs. A recent SMR analysis of workers at three of the plants over an earlier time period in this large company [6] indicated that there was a substantial "healthy worker effect" although the data to do an evaluation of this in our dataset was not available to us. From a graphic comparison of the SMRs from one table in this analysis with our nearest equivalent PCMR results, provided in Figure 1, it appears that the overall pattern is very similar in the two mortality analyses.

Cancer mortality patterns in the general population over the time period 1969–2001 changed somewhat. For example, lung cancer mortality increased dramatically in US females during this time period while cervical cancer mortality decreased. In males, lung cancer mortality increased in the early part of the time period, but began to decline in the 1990s. Accordingly, we examined the workers' mortality experience in five-year time intervals, and we looked at patterns by five-year age groups during these intervals. The excesses of some cancers were greater in the younger age groups and in the 1970s and 1980s rather than the latter years of the study period. These patterns are consistent with a work-related cancer mortality effect that has diminished in recent years.

Additional analyses of specific subgroups of decedents were carried out, primarily because of concerns that toxic exposures used in manufacturing, such as solvents, metals, electromagnetic radiation, and the components of photoresist might be associated with increased cancer mortality. For example, numerous halogenated solvents are used in computer manufacturing [1,2,10] and some of these are associated with increased risk of kidney cancer and cancer of the lymphatic and hematopoietic tissue in workers [8]. No information on individual exposure to specific chemicals or processes was available for this analysis. Accordingly, we examined the mortality patterns among decedents for whom work histories were available and grouped them into manufacturing jobs and presented results for this sub-group.

The overall PMR and PCMR analyses for manufacturing workers revealed a pattern that was similar to the pattern in all workers combined. In most instances, the PMRs and PCMRs that were elevated in the total CMF were somewhat more elevated in the manufacturing workers. The decreased mortality due to respiratory system cancer in males in the CMF was similar in the manufacturing subgroup. However, all the manufacturing subgroup analyses were based on fewer deaths and were therefore statistically less stable. In males employed in manufacturing, there was consistent increased mortality from cancer of digestive organs, kidney cancer, melanoma of skin and cancer of the brain and central nervous system. The PCMRs for several of these cancers were significantly elevated and there was also excess mortality due to non-Hodgkin's lymphoma within the broader category of lymphatic and hematopoietic malignancies. Among female manufacturing workers, we found the breast cancer PMR to be slightly elevated, while the PMRs for kidney cancer and lymphatic and hematopoietic tissue malignancies were significantly elevated but based on few deaths. The female manufacturing workers PCMRs were based on very few deaths and followed a similar pattern.

Mortality analyses such as the one we conducted are limited by the availability of information on death certificates, and typically this does not permit controlling for confounding factors such as prior occupational exposures, non-occupational exposures, or cigarette smoking [14]. Similarly, the cause of death information is often limited and may be incomplete for certain types of cancer. These limitations are well-documented and apply to these CMF analyses, as well. Work history information available for grouping manufacturing workers or locations was also incomplete, but we did not detect any systematic pattern in the missing data that would produce biased estimates of effect. We examined the overall patterns of mortality among those for whom work history information was available and they appeared to be similar to those for whom this information was not available. Consequently, we do not consider the manufacturing analyses to be distorted in any important way; the lack of information reduces the number of deaths available for inclusion in these analyses and therefore reduces the power to detect some associations.

As noted above, this study was conducted in the context of litigation, so we were unable to communicate with the record providers to clarify inconsistencies or fill gaps in the information provided. Here again, we do not consider this to be a significant source of bias, but it limits the power of the sub-group analyses. If additional work is carried out on this database, it would be desirable to do this in an atmosphere of open communication.

## List of Abbreviations

PMR, Proportional mortality ratio

PCMR, Proportional cancer mortality ratio

CI, Confidence Interval

IBM, International Business Machines

CMF, Corporate Mortality File

ICD 9, International Classification of Diseases, 9^th ^revision

OCMAP, Occupational Cohort Mortality Analysis Program

SMR, Standardized Mortality Ratio

## Competing interests

The author was previously paid a consultancy by the plaintiffs' law firm; the law firm did not design or conduct the study, nor review or approve the manuscript. The author received no remuneration for the preparation of this manuscript.

## Authors' contributions

RC was responsible for the overall conception and presentation of the work.
